# Effects of N and P additions on twig traits of wild apple (*Malus sieversii*) saplings

**DOI:** 10.1186/s12870-023-04245-4

**Published:** 2023-05-16

**Authors:** Yuan-Yuan Zhang, Jing-Ming Yan, Xiao-Bing Zhou, Yuan-Ming Zhang, Ye Tao

**Affiliations:** 1 State Key Laboratory of Desert and Oasis Ecology, Key Laboratory of Ecological Safety and Sustainable Development in Arid Lands, Xinjiang Institute of Ecology and Geography, Chinese Academy of Sciences, Urumqi, 830011 Xinjiang China; 2 Xinjiang Key Laboratory of Conservation and Utilization of Plant Gene Resources, Xinjiang Institute of Ecology and Geography, Chinese Academy of Sciences, Urumqi, 830011 Xinjiang China

**Keywords:** *Malus sieversii*, Nutrient addition effect, Growth performance, Plant trait network, Population conservation, Twig trait

## Abstract

**Background:**

Wild apple (*Malus sieversii*) is under second-class national protection in China and one of the lineal ancestors of cultivated apples worldwide. In recent decades, the natural habitation area of wild apple trees has been seriously declining, resulting in a lack of saplings and difficulty in population regeneration. Artificial near-natural breeding is crucial for protecting and restoring wild apple populations, and adding nitrogen (N) and phosphorous (P) is one of the important measures to improve the growth performance of saplings. In this study, field experiments using N (CK, N1, N2, and N3: 0, 10, 20, and 40 g m^− 2^ yr^− 1^, respectively), P (CK, P1, P2, and P3: 0, 2, 4, and 8 g m^− 2^ yr^− 1^, respectively), N20P*x* (CK, N2P1, N2P2, and N2P3: N20P2, N20P4 and N20P8 g m^− 2^ yr^− 1^, respectively), and N*x*P4 (CK, N1P2, N2P2, and N3P2: N10P4, N20P4, and N40P4 g m^− 2^ yr^− 1^, respectively) treatments (totaling 12 levels, including one CK) were conducted in four consecutive years. The twig traits (including four current-year stem, 10 leaf, and three ratio traits) and comprehensive growth performance of wild apple saplings were analyzed under different nutrient treatments.

**Results:**

N addition had a significantly positive effect on stem length, basal diameter, leaf area, and leaf dry mass, whereas P addition had a significantly positive effect on stem length and basal diameter only. The combination of N and P (N*x*P4 and N20P*x*) treatments evidently promoted stem growth at moderate concentrations; however, the N20P*x* treatment showed a markedly negative effect at low concentrations and a positive effect at moderate and high concentrations. The ratio traits (leaf intensity, leaf area ratio, and leaf to stem mass ratio) decreased with the increase in nutrient concentration under each treatment. In the plant trait network, basal diameter, stem mass, and twig mass were tightly connected to other traits after nutrient treatments, indicating that stem traits play an important role in twig growth. The membership function revealed that the greatest comprehensive growth performance of saplings was achieved after N addition alone, followed by that under the N*x*P4 treatment (except for N40P4).

**Conclusions:**

Consequently, artificial nutrient treatments for four years significantly but differentially altered the growth status of wild apple saplings, and the use of appropriate N fertilizer promoted sapling growth. These results can provide scientific basis for the conservation and management of wild apple populations.

**Supplementary Information:**

The online version contains supplementary material available at 10.1186/s12870-023-04245-4.

## Introduction

Wild apple (*Malus sieversii*) is a treasured and endangered species that is under national second-class protection in China. The Tianshan wild fruit forests are mainly distributed in the Tianshan region of Central Asia, and Xinjiang region of China occupies approximately 40% of the total area [1]. Wild apple and *Prunus armeniaca* are the established species in the wild fruit forests, among which wild apple trees form a pure forest [1, 2]. Studies have found that wild apple trees are one of the ancestors of cultivated apple trees and contributed to their evolution [3, 4]. However, in recent decades, the distribution area of wild apple populations in Xinjiang has been drastically shrinking due to various biotic (such as infestation by *Agrilus mali* and *Valsa ceratosperma*, grazing, and logging) and abiotic factors (such as global climate change) [5–7]. In addition, problems such as major habitat fragmentation and reduction of germplasm resources also exist. The natural population is seriously declining and is characterized by a lack of young individuals and a relatively large number of middle-aged and old individuals [5]. Population decline shifts community structure, affects community species composition, inhibits plant productivity, and reduces the rate of litter decomposition and soil nutrient content [6]. Therefore, the regeneration and rejuvenation of wild apple populations need to be urgently addressed.

The growth of trees, especially young trees, is affected by various environmental factors, including interspecific competition, slope direction, soil moisture content, and nutrient limitation [8–10]. Among these, nutrient availability is one of the most critical factors that influences tree survival, productivity, and yield. Based on the differences in soil nutrient status, the appropriate nutrient ratios and amounts of additions required are selected to alleviate nutrient limitation [8, 10, 11]. Previous studies on the effects of nutrient treatments on trees have been focused on simulated nitrogen (N) deposition, phosphorous (P) addition, and mixed N and P addition [10–13]. These studies revealed that the effects of N and P addition on plant growth are inconsistent and may show inhibition, promotion, or no significant effect [10, 13, 14]. Plant species with limited nutrients may be in a disadvantaged position in the competition with other species in the ecosystem, as their growth rate is generally lower than species without limited nutrients [15]. It has been reported that, many more endangered plant species persist under P-limited than under N-limited conditions, that is, enhanced P is more likely to be the cause of species loss than N enrichment. Therefore, nutrient limitation may largely cause species to become endangered [16]. However, different nutrient elements play different roles in this ecological process.

Twigs are among the most active components of the branching system and reflect the plant’s response to the environment better than the whole plant [17, 18]. The twigs consist of two components—stems and leaves. Stems mainly provide mechanical support and ensure transport, and leaves mainly perform photosynthesis. Stems and leaves show a general correlation and variability between them as they adapt to the environment through combinations and trade-offs of their functional traits, thereby determining the ecological strategy of the plants [18, 19]. Corner was the first to propose that size and number of twig leaves are dominant dimensions reflecting plant life history strategies [20, 21]. Nutrient addition effects on plant growth vary with species and plant traits [19, 20]. In general, N additions usually showed inhibition at high concentration and promotion at low concentrations of plant biomass. Compared with N and N + P treatments, P addition increased tree growth in most cases, especially in low latitude areas [10]. The “growth rate hypothesis” proposes that when an organism has a higher growth rate, it also has a higher P content and a lower N:P [22]. Through the study on the nutrient addition of apple (*Malus hupehensis*) seedlings, it was found that the effect of N and P mixed addition and phosphorus addition was significantly higher than that of N addition alone [23]. Combined application of N and P significantly improved the growth of apple rootstock seedlings [24]. Taken together, most studies focused on the individual effects of N and P addition on plant growth, and studies on the effects of combined N and P treatment on twig traits are still insufficient.

Currently, the twig traits of wild apple saplings and their interactions in response to exogenous N and P applications are unclear, which is detrimental to the conservation and sustainable use of this endangered plant. A latest report has revealed that N is more susceptible to the stressing for adult wild apple trees in natural population, especially for the declining trees [18]. Because young trees are more sensitive to external environmental changes than adult woody plants [14], we hypothesized that adding N rather than P had stronger impacts on twig traits of wild apple saplings, and the combined N and P addition exhibited more positive effect on plant performance; however, the response levels of different traits were different. In this study, field experiments on nutrient addition to wild apple saplings, including N, P, N20P*x* (i.e., fixed N concentration with variable P concentration) and N*x*P4 (i.e., variable N concentration with fixed P concentration) treatments (totaling 12 treatment levels) were conducted for four consecutive years (2017–2020). The objectives of this study were (1) to clarify the response strengths of stem and leaf traits of wild apple saplings under different N and P treatments and (2) to explore the differences in twig trait associations and the comprehensive growth performance of wild apple saplings under N and P treatments. The results will provide a basis for predicting the growth of wild apple saplings under exogenous nutrient application, thereby helping in the regeneration and rejuvenation of wild apple populations and scientific management.

## Materials and methods

### Study area

The study site is located in the Gailiangchang, Xinyuan County, Ili Autonomous Prefecture (83°36’ E, 43°22’ N; alt. 1027 m), and receives an annual mean precipitation of 400–500 mm [9]. The region belongs to the temperate continental semi-humid desert climate zone. The annual mean temperature in the study area is 6–9.3 °C, the snow period is for 150 d, the frost-free period is for 140–180 d, and the annual sunshine duration is 2500 h [5, 9]. Besides, the annual atmospheric N deposition rate is approximately 0.8 g N m^− 2^ a^− 1^ [25, 26]. In winter, the cold air in the Ili Valley sinks to the valley floor resulting in the formation of a relative temperature layer on the mountain slopes, which is called the “inversion layer”. This protects plants from the severe cold and enables them to overwinter smoothly [27]. The soil mostly developed on loess or parent materials and was characterized by thick humus, moderate and loose structure, high porosity, but in some areas, the soil nutrient content is relatively low (e.g., Gongliu County) [18, 28], Ili region is a “refuge” for wild apple trees and other flora in Xinjiang because of the unique climatic and environmental conditions.

### Experimental design

In October 2016, the experimental site was pre-treated by performing land leveling and delineation of sampling plots. Soil sample (0–10 cm, five replicates) was collected to determine the physical and chemical properties of the soil (Table 1). In early April 2017, the experimental sample plots were constructed before the germination of wild apple seedlings, and they were set up using a completely randomized design (Fig. S1). Soil pH are basically consistent with previous studies, the pH of the soil where wild apple saplings grow is generally neutral and slightly alkaline [18].

**Table 1:** Soil physical − chemical properties of the experimental plots for wild apple saplings

Item	SOC (g kg^− 1^)	TN (g kg^− 1^)	TP (g kg^− 1^)	AN (mg kg^− 1^)	AP (mg kg^− 1^)	pH	EC (µS cm^− 1^)
Mean ± S.E.	29.850 ± 1.341	2.473± 0.031	0.994 ± 0.022	130.796± 7.184	5.408± 0.582	8.075 ± 0.021	199.320 ± 28.29

SOC: soil organic carbon; TN: total soil N; TP: total soil P; AN: soil available N; AP: soil available P; pH; EC: soil electrical conductivity

Based on nutrient additions in the surrounding apple orchards, literature research, and the actual status of soil nutrient here, the N treatments were as follows: 10, 20, and 40 g m^− 2^ yr^− 1^; the P treatments were as follows: 2, 4, and 8 g m^− 2^ yr^− 1^ [29, 30]. Combined N and P treatments were of two types as follows: first, a medium N concentration with three different P concentration ratios: N20P2, N20P4, and N20P8; second, three different N concentrations with a medium P concentration ratio: N10P4, N20P4, and N40P4, where both treatments have N20P4 in common. The four nutrient treatments shared a control group (CK), and the CK consisted of the same amount of water. These fertilization treatments were applied to a total of 12 groups, with five replicates for each treatment level. The total number of plots was 60, and size of each plot was 1.5 × 1.5 m (Fig. S2). Each plot was labeled with a card. The periphery of the sample square was raised to prevent interference among sample squares, with a minimum distance of 1 m between plots.

The transplanted wild apple seedlings were locally cultivated one-year-old trees, and the seeds were collected from the same population to ensure consistent genetic background and sapling growth. Wild apple seedlings were planted at a distance of 0.3 m from the four diagonal corners of the sample square to ensure that the four trees had relatively sufficient growing space.

N and P were supplied as CON_2_H_4_ and Ca(H_2_PO4)_2_·CaHPO_4_, respectively, which are most commonly used in agroforestry. To apply the fertilizer, pits of approximately 20 cm in depth and 15 cm in diameter were dug at distance of 0.3 m from the periphery of the plots (Fig. S2). The fertilizer was then evenly applied in the four pits at the diagonals of each plot and 200 mL of water was added, followed by filling and covering with soil to promote nutrient uptake by the roots. Saplings were quickly pruned and watered after transplanting to ensure their rapid branching and survival. Fertilizer was applied twice a year—at the end of April (leaf budding period) and the beginning of October (leaf-falling period)—and fertilization treatments were conducted for four consecutive years (2017–2020) [26, 31]. Normal field management was conducted at the experimental site, with no artificial watering throughout the year to approximate natural conditions, except during fertilization.

### Twig sampling and trait measurement

The current-year twig samples were collected in August 2020 during the peak growing season for stem and leaf. In this study, current-year twigs without branching, flowers and fruits at the terminals, consisting of the apical internode (referred to as stem hereafter) and the leaf it bore, and without the loss of any leaf, were defined as twigs [17, 32, 33].When sampling, wild apple saplings were five years old, with a mean tree height of 2.14 m and a basal diameter of 2.24 cm. Except for a very few saplings that bore a very few fruits, most saplings had still not entered the reproductive stage. When collecting twigs, the main branches were avoided, and medium-sized current-year lateral branches in the middle of the canopy were selected [34, 35]. These branches were cut off with branch shears from their base and transported to the laboratory in labeled envelopes. For each tree, current-year twigs (*n* = 1–3) with similar size were collected from the medium sunny position of the tree canopy.

The stems and leaves were separated and the length (L; in cm) of the stem was measured with a straightedge, the basal diameter (BD; in cm) of the stem was measured with a vernier caliper, the leaves were scanned with a scanner (CanoScan LIDE120, Canon, Japan), and the number of leaves (leaf number, LN) on the stem was counted and recorded. The samples were oven-dried at 70 °C to constant weight, and the total blade mass (TBM; in g), total petiole mass (TPM; in g), stem mass (SM; in g), and twig mass (TM = TBM + TPM + SM; in g) were measured using an analytical balance (accurate to 0.0001 g). The total leaf area (TLA; in cm^2^) was measured using a software program (LA-S, Wanshen, Hangzhou, China). The above-mentioned indicators were used to calculate twig and leaf-related indicators, including total leaf mass (TLM = TBM + TPM; in g), single petiole mass (SPM; in g), single blade mass (SBM; in g), single leaf mass (SLM = SPM + SBM; in g), single leaf area (LA; in cm^2^), specific leaf area (SLA; in cm^2^ g^− 1^), leafing intensity (LI = leaf number/SM; in n g^− 1^), leaf area ratio (LAR = total LA/TM; in cm^2^ g^− 1^), and leaf area-to-stem mass ratio (LAMR = total LA/SM; in cm^2^ g^− 1^).

### Statistical analyses

The normality of all data was checked by conducting the K-S test; all data followed a normal distribution. The effects of adding N and P and their interaction effects on twig traits of wild apple trees were examined by performing one-way ANOVA and two-way ANOVA, and multiple comparisons were performed using Duncan’s method (*α* = 0.05). The effects of the four nutrient treatments on the twig traits were calculated using Eqs. 1–6. (1) The addition of only N (without adding P; N10, N20, and N40 vs. CK); (2) the addition of only P (without adding N; P2, P4, and P8 vs. CK); (3) N20P*x* (N20P2, N20P4, and N20P8 vs. CK); (4) N*x*P4 (N10P4, N20P4, and N40P4 vs. CK); (5) the effect of P with the addition of N (N20P2, N20P4, and N20P8 vs. N20); (6) the effect of N with the addition of P (N10P4, N20P4, and N40P4 vs. P4). The values represented means with 95% confidence intervals (CIs). The effect is considered to be significant (*) when the 95% CI does not overlap with zero.

1 $$dN=\frac{YN - YCK}{YCK }\times 100\text{\%}$$

2 $$dP=\frac{YP - YCK}{YCK }\times 100\text{\%}$$

3 $$dN20Px=\frac{YN20Px - YCK}{YCK }\times 100\text{\%}$$

4 $$dNxP4=\frac{YNxP4 - YCK}{YCK }\times 100\text{\%}$$

5 $$\begin{gathered} dP\,effect\,with\,N\,addition\, =  \hfill \\\,\frac{{YN20Px - YN20}}{{YN20}} \times 100\%  \hfill \\ \end{gathered} $$

6 $$\begin{gathered} dN\,effect\,with\,P\,addition\, =  \hfill \\\,\frac{{YNxP4 - YP4}}{{YP4}} \times 100\%  \hfill \\ \end{gathered} $$

Here, *N* indicates N10, N20, and N40; *P* indicates P2, P4, and P8; *N20Px* indicates N20P2, N20P4, and N20P8; *NxP4* indicates N10P4, N20P4, and N40P4; CK indicates the control.

Plant trait networks (PTNs) were designed using the R software (version 4.2.1) to determine the relationship among several twig traits and their significance. PTNs can be used to visualize complex relationships among traits, clarify the “dimensions” of multiple trait composition, quantify mutual dependencies among multiple traits, identify key traits, and compare complex relationships among traits in different environments [36]. First, the correlation coefficients (r) were determined by performing Spearman’s correlational analysis using the ‘psych’ R package, and the significant correlations between twig traits (*P* < 0.05) were retained to avoid spurious correlations between traits [36–38]. Second, the differences in network structures were compared using topological parameters of PTNs, including degrees, mediators, clustering coefficients, edge densities, and modularity [36, 38]. Finally, the effect of adding N and P on the comprehensive resistance of twig traits was analyzed using the membership function in fuzzy mathematics (7 and 8) [39]. The formula for the membership function is shown in Eq. (7):

7 $$\text{U}\left(Xij\right)=\frac{Xij - Xjmin}{Xjmax - Xjmin }$$

If the parameters were negatively correlated, the conversion was performed using the inverse membership function (Eq. 8), and the formula was calculated as:

8 $$\text{U}\left(Xij\right)=1 - \frac{Xij - Xjmin}{Xjmax - Xjmin }$$

Here, U(*X*_*ij*_) denotes the membership function value of trait j in treatment i; *X*_*ij*_ denotes the measured value of trait j in treatment i; *X*_*jmax*_ and *X*_*jmin*_ denote the maximum and minimum measured values of the indicators in each trait, respectively.

The PTNs and their topological parameters were visualized using the ‘igraph’ R package and Cytoscape 3.8.0. The data were statistically analyzed using SPSS 26.0 (SPSS Inc., Chicago, Illinois, USA), and graphs were made using OriginPro 2021.

## Results

### Variation in current-year stem traits

**Fig. 1:**
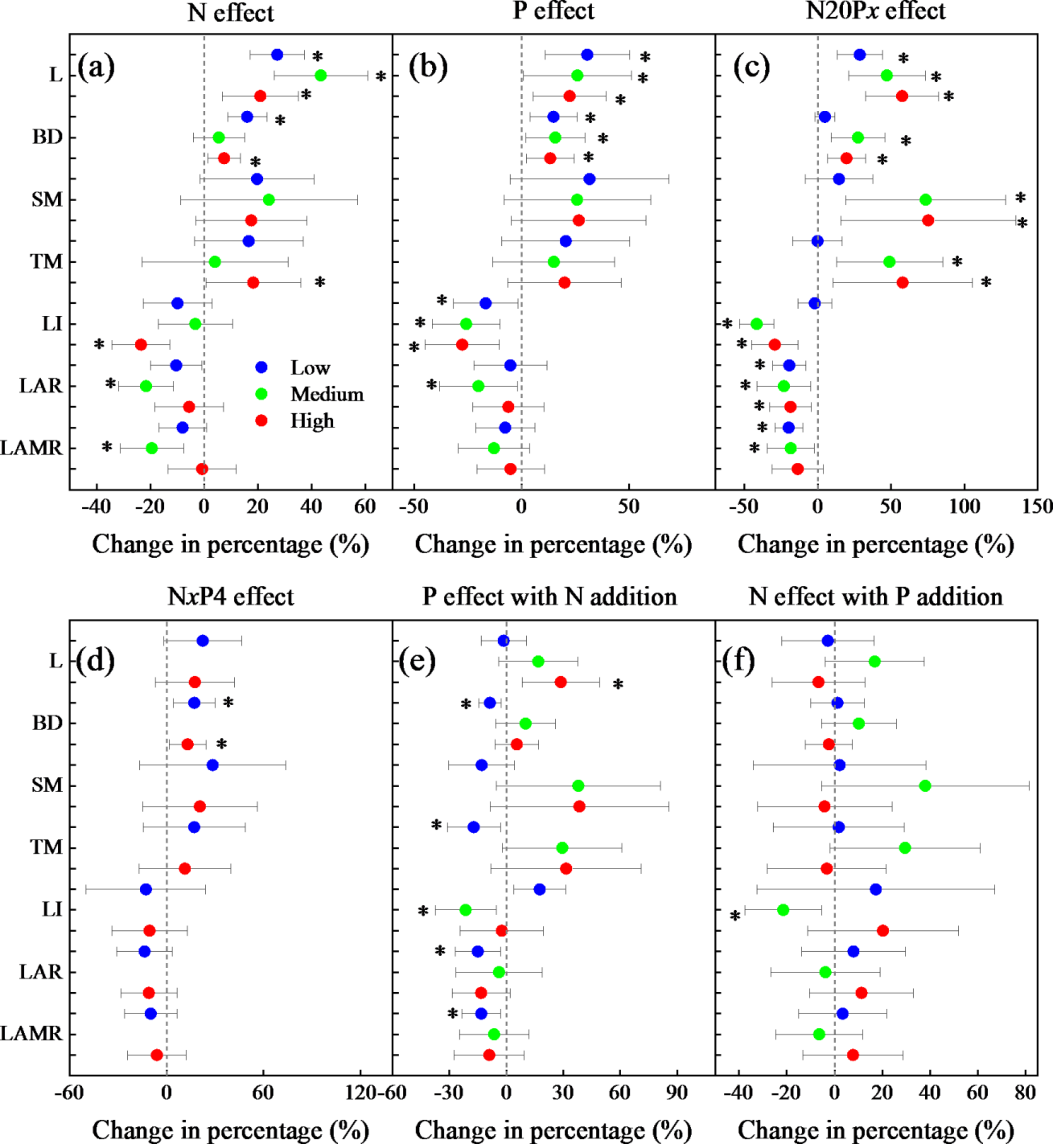
The effects of *Malus sieversii* stem traits for treatment with N, P, and N + P. The effects of treatment with only N (without P addition), only P (without N addition), N20P*x*, N*x*P4, P with the addition of N, and N with the addition of P on stem length (L), basal diameter (BD), stem mass (SM), twig mass (TM), leaf intensity (LI), leaf area ratio (LAR), and leaf area-to-stem mass ratio (LAMR) of *Malus sieversii*. The values represent the mean with 95% confidence intervals (CIs). The effect was considered to be significant (*) when the 95% CI did not overlap with zero. Low: N10, P2, N20P2, and N10P4; Medium: N20, P4, and N20P4; High: N40, P8, N20P8, and N40P4. N20P4 is common to both N20P*x* and N*x*P4, therefore, it is only shown in the former. (**a**): N effect, (**b**): P effect, (**c**): N20P*x* effect, (**d**): N*x*P4 effect, (**e**): P effect with N addition, (**f**): N effect with P addition. the same below

The addition of only N significantly (*P* < 0.05) increased L (length) at all concentrations, increased BD (basal diameter) considerably at low and high concentrations, and TM (twig mass) at high concentrations, but significantly decreased LAR (leaf area ratio) and LAMR (leaf area-to-stem mass ratio) at medium concentrations and LI (leafing intensity) at high concentrations (Fig. 1 and S3). The addition of only P significantly increased L and BD at all concentrations but decreased LI considerably at all concentrations and LAR at medium concentrations. Treatment with N20P*x* significantly increased L at all concentrations and increased BD, SM (stem mass), and TM at medium and high concentrations but decreased LI and LAR substantially at all concentrations and LAMR at low and medium concentrations. Treatment with only N*x*P4 significantly promoted BD at all concentrations, while adding P showed a highly negative effect of N (i.e., N + P addition vs. P addition) only for medium concentrations on LI. The addition of N showed a highly positive effect of P (i.e., N + P addition vs. N addition) only for high concentrations of L, while a substantial negative N effect was observed for medium concentrations of LI and low concentrations of BD, TM, LAR, and LAMR. Overall, adding only N or P had significant positive effects on L and BD and showed different degrees of negative effects on the indicators in the stem-to-leaf trait ratios. Treatment with N20P*x* had a more prominent positive and negative effect on stem traits and the ratio of stem-to-leaf traits at all concentrations, and treatment with N*x*P4 showed a significant positive effect on stem traits at medium concentrations.

### Variation in leaf traits

**Fig. 2:**
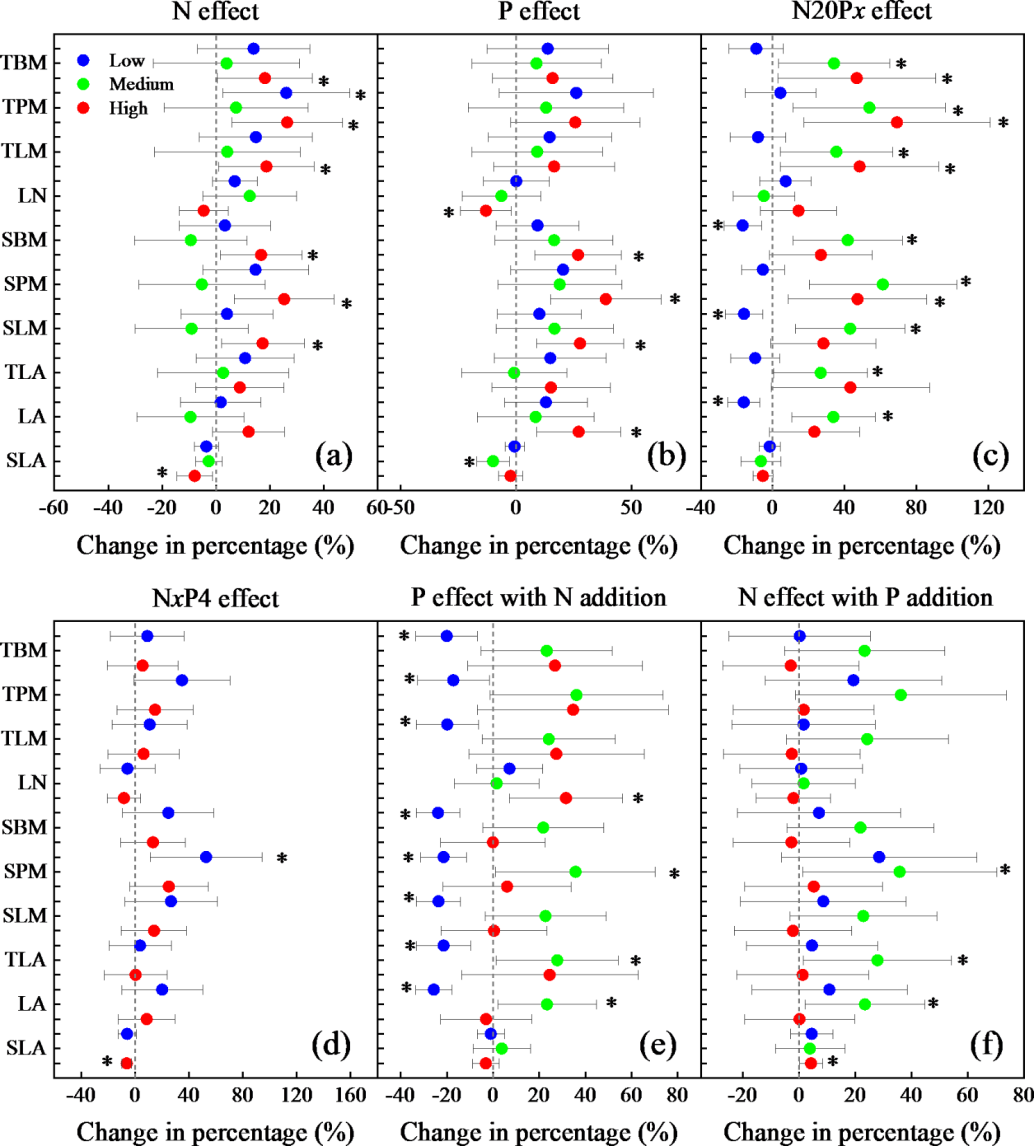
The effects of *Malus sieversii* leaf traits for treatment with N, P, and N + P. The effects of only N (without P addition), only P (without N addition), N20P*x*, N*x*P4, P with the addition of N, and N with the addition of P on leaf area (LA), leaf number (LN), total blade mass (TBM), total petiole mass (TPM), total leaf area (TLA), total leaf mass (TLM), single petiole mass (SPM), single blade mass (SBM), single leaf mass (SLM), and specific leaf area (SLA) of *Malus sieversii*. The values represent the mean with 95% confidence intervals (CIs). The effect was considered to be significant (*) when the 95% CI did not overlap with zero. Low: N10, P2, N20P2, and N10P4; Medium: N20, P4, and N20P4; High: N40, P8, N20P8, and N40P4. N20P4 is common to both N20P*x* and N*x*P4, therefore, it is only shown in the former

Adding only N significantly (*P* < 0.05) increased TPM (total petiole mass) at low concentrations, TBM (total blade mass), TPM, TLM (total leaf mass), SBM (single blade mass), SPM (single petiole mass), and SLM (single leaf mass) at high concentrations and decreased SLA (specific leaf area) considerably at high concentrations (Fig. 2 and S4). Adding only P significantly increased LA (leaf area), SBM, SPM, and SLM at high concentrations but significantly decreased LN (leaf number) at high concentrations and SLA at medium concentrations. Adding N20P*x* considerably increased total leaf dry mass (TBM, TPM, and TLM), SPM, TLA (total leaf area), SBM, and LA at medium concentrations and significantly decreased SBM, SLM, and LA at low concentrations. Treatment with N*x*P4 significantly increased SPM at low concentrations and significantly decreased SLA at high concentrations. Applying N had a significant positive effect of P (i.e., N + P addition vs. N addition) on SPM, TLA, and LA at medium concentrations and SLA at high concentrations. Applying P had significant positive effects of N (i.e., N + P addition vs. P addition) on SPM, TLA, and LA at medium concentrations and LN at high concentrations, while it had significant negative effects of N on total leaf mass (TBM, TPM, and TLM), single leaf mass (SBM, SPM, and SLM) and leaf area (LA and TLA) at low concentrations. The changes in nutrients differed across different N and P treatment levels, and adding high levels of N showed a positive effect on leaf dry mass (total leaf mass and single leaf mass). The addition of N20Px promoted most leaf traits at medium-to-high concentrations. Except for LN and SLA, the effect of P with the addition of N showed inhibition at low concentrations.

### Twig trait networks and the membership function values under the effects of N and P

**Fig. 3:**
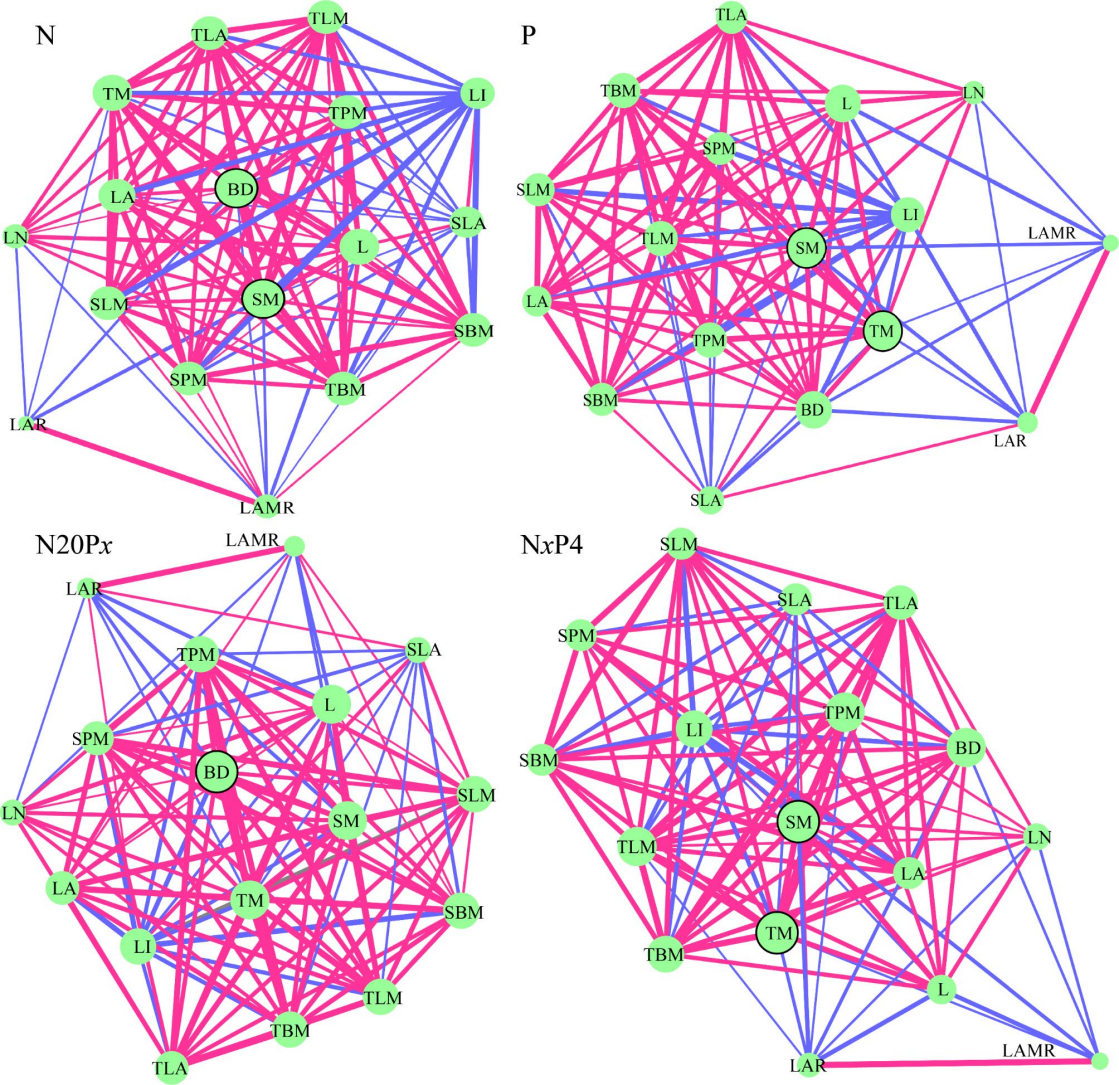
Plant trait networks (PTNs) of *Malus sieversii* for treatment with N, P, and N + P. Nodes indicate different twig traits, and node size indicates the degree. Red and blue lines indicate positive and negative correlations, respectively. Circles with black borders represent the key trait. The width of the line indicates the strength of the association. L: Length; BD: basal diameter; LA: leaf area; LN: leaf number; TM: twig mass; TBM: total blade mass; TPM: total petiole mass; SM: stem mass; TLA: total leaf area; TLM: total leaf mass; SPM: single petiole mass; SBM: single blade mass; SLM: single leaf mass; SLA: specific leaf area; LI: leaf intensity; LAR: leaf area ratio; LAMR: leaf area-to-stem mass ratio. The topological properties of the network and the characteristics of the node (trait) are shown in Tables S1 and S2. N20P*x*: N20P2, N20P4, and N20p8; N*x*P4: N10P4, N20P4, and N40P4

The topology parameters of twig trait networks differed significantly (*P* < 0.05) under the effects of N and P treatments (Fig. 3 and Table S1). For topological parameters, the PTNs for treatment with P and N*x*P4 showed a lower edge number, connectedness, and average degree than the PTNs for treatment with N and N20P*x*, while the average path, centralization degree, and modularity of the PTNs for treatment with P and N*x*P4 were higher than those of the PTNs for treatment with N and N20P*x*.

The network node parameters were used to identify key traits, which differed with the addition of nutrients (Fig. 3 and Table S2). The results showed that BD and SM had the highest degree (16) when only N was added, and they regulated the wild apple phenotype; L and TM (degree = 15) also influenced the PTN. Additionally, BD and SM had the highest betweenness (3.541), which indicated that they strongly affected the multifunctional coupling relationships. Under P treatment, SM and TM showed the highest degree (16) and betweenness (4.81), followed by L and BD, which showed the second highest degree (15). Also, BD had the highest degree (16) and betweenness (3.63) under N20P*x* treatment, followed by SM and TM, which had the second highest degree (15). SM and TM showed the highest degree (16) and betweenness (5.106) under N*x*P4 treatment, followed by BD, TPM, and TLM, which had the second highest degree (15). The PTN analysis of all data showed that BD, SM, TM, and LA were the key traits with the highest degree (16) and betweenness (1.37). Thus, prominent differences were observed in the key traits of the PTNs under nutrient treatments, but the key traits could coordinate multiple functional coupling while regulating the phenotype.

**Fig. 4:**
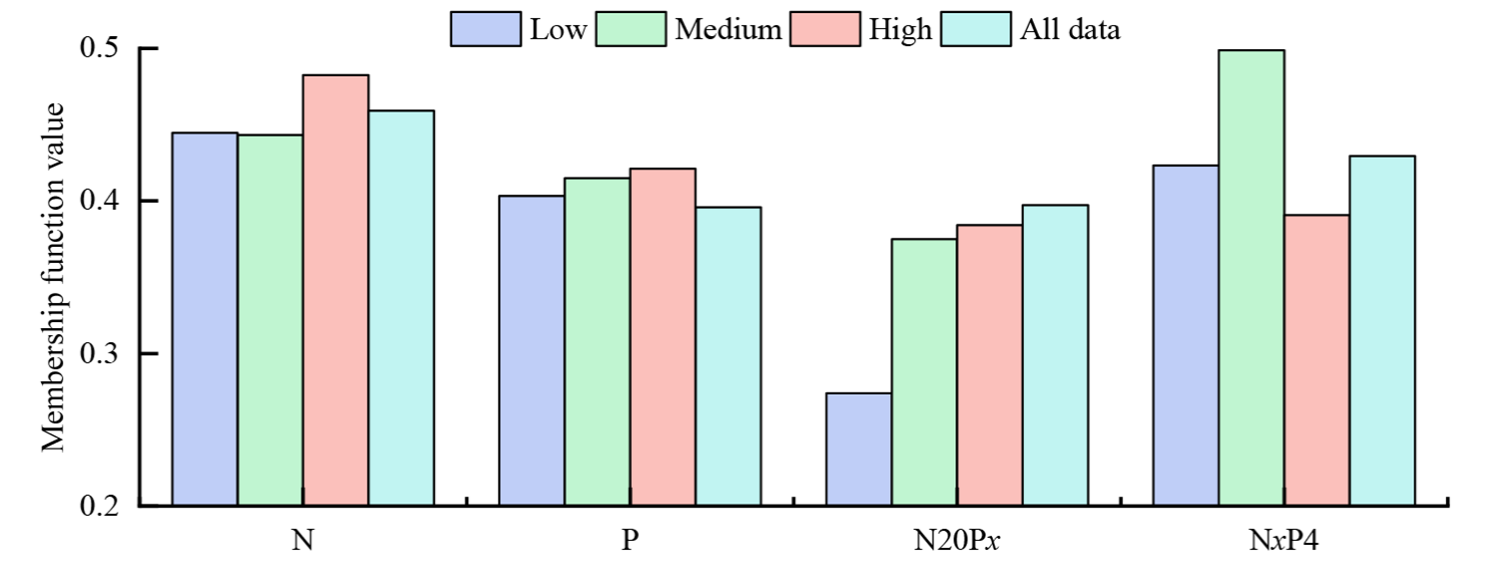
The membership function values of twig traits of wild apple saplings treated with N, P, N20P*x*, and N*x*P4. Low: N10, P2, N20P2, and N10P4; Medium: N20, P4, and N20P4; High: N40, P8, N20P8, and N40P4

The results of the membership function showed that the membership function values of all twig traits for different N and P treatments followed the order N > N*x*P4 > N20P*x* > P (Fig. 4). This indicated that the application of N alone or N with an appropriate amount of P (i.e., N*x*P4; except for N40P4) improved the comprehensive growth performance of wild apple saplings.

## Discussion

### Effect of adding N and P on stem traits of wild apple saplings

Our results indicated that adding medium concentrations of N significantly (*P* < 0.05) increased twig L (Table 1; Fig. S3). Twig L and BD were strongly and positively affected by the addition of N and P (all concentrations), but TM was not significantly affected (Fig. 1). These findings were similar to those of a study on the effect of the addition of N and combined N and P on the two kinds of *Larix* sapling [40]. Other studies have shown that the growth of wild apple trees is N-limited and that N application can alleviate nutrient limitation, possibly leading to a shift in resource utilization by wild apple trees [9, 18, 41, 42]. The amount of P in plants also affects their growth rate. Plants need large amounts of N and P to meet their growth requirements, especially in actively growing saplings [43]. The enlarged stems of twigs contain larger vessels, which facilitate the transport of nutrients and water, while longer stems help plants obtain resources such as sunlight [44, 45].

The effect of the combined addition of N and P on plant growth depends on the dynamic balance between soil nutrient supply and plant nutrient requirements. However, the combined effect is usually better than that of the addition of only N or P [46–48]. The addition of N increases the activity of P by decreasing the pH, which in turn increases the effectiveness of soil P and promotes the uptake of limiting nutrients by plants [41]. However, the differences in the response of plants to the combined addition of N and P are attributed to factors such as nutrient limitation and plant species [38, 47, 49]. In the present study, the combined treatment of N and P (N20P*x* and N*x*P4) promoted twig traits (Fig. S3). For the effect of the interaction between N and P (Fig. 1), the addition of N20P*x* showed significant positive effects on L, BD, and dry weight at medium and high concentrations; also, the N*x*P4 treatment showed significant positive effects on BD at all concentrations. These findings were similar to those of a study by Cheng et al. (2022) who studied the response of *Machilus pauhoi* to the addition of N and P [50]. Adding N significantly increased stem diameter under the combined treatment of N and P (i.e., the effect of P with the addition of N), and the effect was higher than the effect of adding P (i.e., the effect of N with the addition of P) on stems [44]. Regarding stem traits, the size of the stem increased mainly because of an increase in the radial thickness of vascular bundles and the cortex [44]. The increase in BD indicated that the addition of nutrients improved the efficiency of the transport of water and mineral in plants and maintained the balance between twig growth and nutrient transport [44]. The results of the combined treatment of N and P (Table 1) showed that the low concentration of N20P*x* and the effect of P with the addition of N significantly inhibited the stem traits, while high concentrations (low N:P) significantly and positively affected the stem traits [46]. These findings indicated that high N:P inhibited stem growth, while low N:P promoted stem growth. This was consistent with the “growth rate hypothesis”, i.e., low N:P enhances plant growth rates [43].

### Effect of adding N and P on leaf traits of wild apple saplings

In this study, the addition of N and P had no significant effect on leaf traits (Fig. S4). Our findings were similar to those of a study by Mo et al. (2020) on tropical trees, where the researchers showed that adding N and P did not significantly alter leaf traits [51]. They attributed this phenomenon to three factors. First, leaf traits are relatively conserved during development. Second, atmospheric N deposition counteracts the effects of the addition of experimental N [25, 26]. Third, leaves maintain constant values as an adaptation to P-deficient environments [51]. The effects of the addition of only N or P showed (Fig. 2) that adding N positively affected leaf dry mass, while adding P did not significantly affect leaf traits (except for total leaf area and leaf area). This probably occurred because plant growth in this study was N-limited. Our results confirmed to some extent that plant growth in mountainous areas is usually N-limited, rather than P-limited [52, 53].

Some studies have shown that the effect of the combined addition of N and P on plant traits is higher than the effect of adding only N or P [46]. The specific leaf area (SLA) reflects the ability of leaves to capture light and is also a key indicator of plant growth and resource acquisition and conservation strategies [54]. In this study, SLA decreased significantly at medium concentrations of P (*P* < 0.05) (Fig. S3), but the decrease in SLA at other concentrations was not significant. Most experiments attributed the changes in SLA to the degree of nutrient limitation, light effectiveness, and tree species [54, 55]. Additionally, interactions among the availabilities of N, P, and other elements can lead to confusion regarding the effects of soil nutrient availability on SLA [55]. Moreover, the leafing intensity decreased significantly after adding N and P, while the number of leaves did not decrease significantly (Fig. 1 and S4), indicating that saplings of deciduous species invest more resources in stems after the addition of nutrients, and longer current-year twigs facilitate canopy expansion and space acquisition [18, 41].

The combined addition of N and P significantly increased the leaf dry mass (Fig. 1), and these changes were similar to those of the dry mass of the stem in twigs. Treatment with N20P*x* at medium to high concentrations showed significant positive effects on leaf dry mass and leaf area, indicating that adding medium concentrations of N alleviated the effects of the addition of P on some leaf cellular and physiological structures and promoted protein synthesis [56]. This result was consistent with that of a study by Avolio et al. (2014) who reported that the combined addition of N and P on net primary productivity has a stronger effect than the addition of only N or P, probably because the addition of only N or P aggravates the limitation by other nutrients, while the combination of N and P promotes plant growth [57, 58]. In the present study, the effect of N with the addition of P and the effect of P with the addition of N also demonstrated this effect. The application of N had a significant positive effect of P on leaf dry mass and LA. Consequently, the application of P had a significant positive effect of N on LA, SPM, and TLA at medium concentrations.

### Variations in twig trait networks and the growth performance of wild apple saplings after adding N and P

PTNs are a new tool for investigating complex relationships among multiple plant traits [36, 38]. Such complex relationships arise because most traits are not independent of each other, and potential trade-offs and coordination between traits might allow plants to adapt and respond to environmental changes and enhance plant survival [38]. As plant traits perform the same function together, adaptation across resource gradients results in a complex network of trait correlations [36, 59]. The parameters of PTNs can be used to quantify the complex relationships between traits. When a trait is more tightly connected to other traits, it might have greater importance in regulating plant functions [38, 59]. Research showed that leaf thickness is central to the trait network and strongly regulates the stability of the PTN, as determined by investigating the leaves of tropical to temperate trees in eastern China [60]. A study that investigated different water TP levels found that a high level of TP decreased the connectivity of a large PTN and plant functional potential [51]. In this study, in addition to the key traits included LA in the overall data, the key traits of the twig trait network with the addition of N and P were all related to the stem traits (BD, SM, and TM) (Fig. 3 and S5; Table S2). This indicated that stem traits strongly influence the construction of plant functions and the regulation of resources, and are crucial for the stability of the twig trait network. The traits BD, SM, and TM are necessary for nutrient transport, mechanical support, and spatial expansion of plants, respectively [21, 32]. These findings were similar to those of a study by Kleyer et al. (2019) on herbaceous trait networks where stem mass and stem-specific length were found to be the “hub” trait [37]. Li et al. (2021) showed that broad-leaved species have a stronger network than coniferous species, and they might also be more efficient than coniferous species [38]. Besides, the combined addition of N and P increased the stability, closeness, and connectivity of the trait network of *Alyssum linifolium*, which facilitated the growth and function of ephemeral plants [61]. In this study, we showed that the addition of N improved the coordination, closeness, and connectivity of PTNs compared to the addition of P, N20P*x*, and N*x*P4. This largely indicated that adding N changed the ratios of soil nutrients and increased the effectiveness of soil nutrients, which enhanced plant nutrient uptake and promoted stronger connections in the PTNs [46, 61]. The network clustering coefficients also showed the same conclusion that clustering coefficients were lower after adding N than after adding P, N20P*x*, and N*x*P4. But the modularity was higher after adding N than after adding P, N20P*x*, and N*x*P4, which indicated that with the combined addition of N and P, wild apple saplings could perform a specific function with fewer resources invested [38, 60].

The comprehensive growth performance was evaluated after adding N and *P* using the membership function in fuzzy mathematics [39]. The membership function analysis is a method based on the determination and analysis of multiple trait indicators, followed by a comprehensive evaluation of the growth performance of plants [39]. The method is based on the weighted mean of several measurement traits for a more comprehensive and systematic assessment of each trait, which can take many factors into account for evaluating growth performance more objectively, comprehensively, concisely, and accurately [39]. Membership functions are used to comprehensively evaluate forage germplasm resources, peanut germplasm resources, and plant resistance [5, 39], among others. A study showed that biomass and plant tissue N content of Gramineae can be increased by adding N in N-limited plateau regions [62]. Similar results were found for shrubs in coastal areas [43]. The increase in P stocks was greater after adding N and P compared to that after adding only N in the aboveground parts of herbaceous plants [63]. When plants are grown in a nutrient-limited environment, adding N or a certain proportion of P artificially can effectively improve the growth capacity of the plants [21, 46]. The results of this study showed (Fig. 4) that the improvements in twig traits followed the order N > N*x*P4 > N20P*x* > P, indicating that the application of N (N only) or a certain amount of P (N*x*P4; except for N40P4) improved the comprehensive growth performance of wild apple saplings, which was similar to the results of another study [21, 46]. This result will help to provide scientific basis for the management of wild apple saplings in the Tianshan Mountains.

## Conclusions

Based on the four-year field experiments involving the addition of nutrients, the effects of adding only N, only P, and N + P on twig traits of wild apple saplings were systematically analyzed. Adding only N showed significant positive effects on twig stems and leaves; adding only P showed highly positive effects on several twig traits; the effect of the combined addition of N and P on twig traits differed depending on the concentrations and ratios of N and P added, but significant improvements were detected for most twig traits. In the PTNs, twig stem traits (BD, SM, and TM) were the key traits that were influenced by the addition of N and P, corroborating the key position of stem traits in phenotype construction. The membership function showed that adding only N or N with a certain amount of P (N*x*P4) improved the adaptive capacity of wild apple trees; N alone had a better promotion effect on growth performance. These results suggested that applying nutrients while planting wild apple tree seedlings can improve the environmental adaptability and growth status of wild apple saplings. Our findings might contribute to the conservation and scientific management of the rare and precious germplasm resources of wild apple trees.

## Electronic supplementary material

Below is the link to the electronic supplementary material.

Supplementary Material 1

Supplementary Material 2

## Data Availability

The original contributions presented in the study are included in the article/supplementary material. Further inquiries can be directed to the corresponding author.
