# Supplementary material for: Effects of N and P additions on twig traits of wild apple (Malus sieversii) saplings
Source: BMC Plant Biol. 2023 May 16;23:257. doi: 10.1186/s12870-023-04245-4 (PMC10186701; doi:10.1186/s12870-023-04245-4)
Supplement: Supplementary file 1 — Supplementary Material 1 [file 12870_2023_4245_MOESM1_ESM.docx]

**Effects of N and P additions on twig traits of wild apple (*Malus sieversii*) saplings**

**Yuan-Yuan Zhang^1,2,a^, Jing-Ming Yan^1,2,a^, Xiao-Bing Zhou^1,2^, Yuan-Ming Zhang^1,2^, Ye Tao^1,2,*^**

^1^State Key Laboratory of Desert and Oasis Ecology, Key Laboratory of Ecological Safety and Sustainable Development in Arid Lands, Xinjiang Institute of Ecology and Geography, Chinese Academy of Sciences, Urumqi 830011, Xinjiang, China.

^2^Xinjiang Key Laboratory of Conservation and Utilization of Plant Gene Resources, Xinjiang Institute of Ecology and Geography, Chinese Academy of Sciences, Urumqi 830011, Xinjiang, China.

*** Correspondence:**

Ye Tao

[taoye@ms.xjb.ac.cn](mailto:taoye@ms.xjb.ac.cn)

^a^Yuan-Yuan Zhang and Jing-Ming Yan contributed equally to this work and share the first authorship.

**Supplementary Figures and Tables**

**Supplementary Figures**


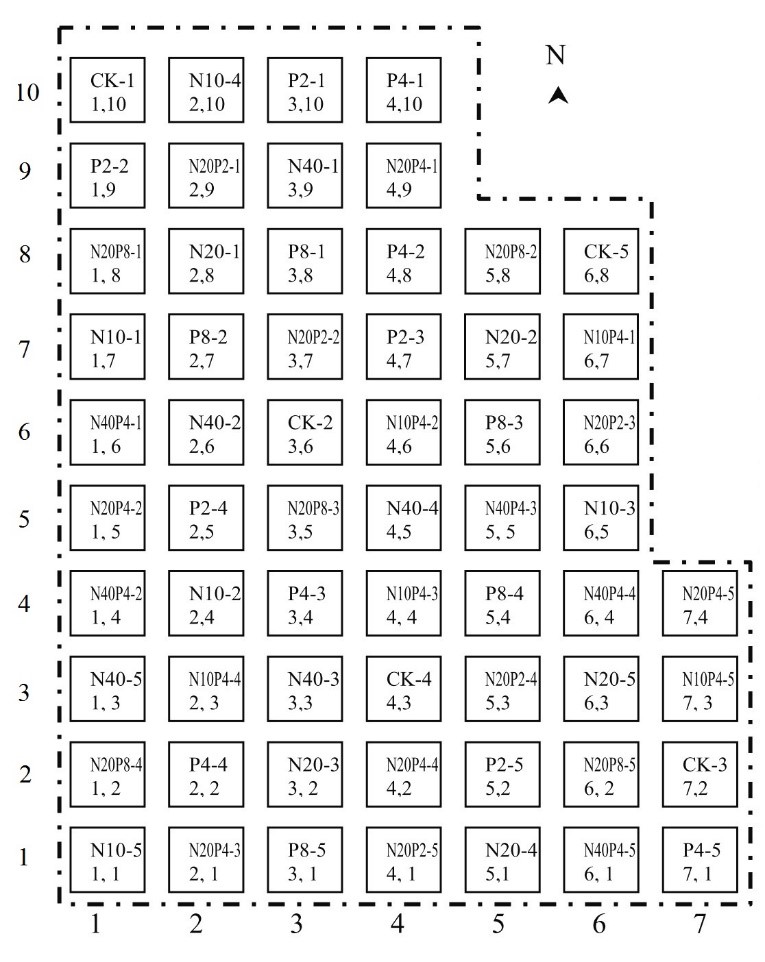


**Fig. S1** Plot distribution for wild apple sapling under N, P, and N+P additions.


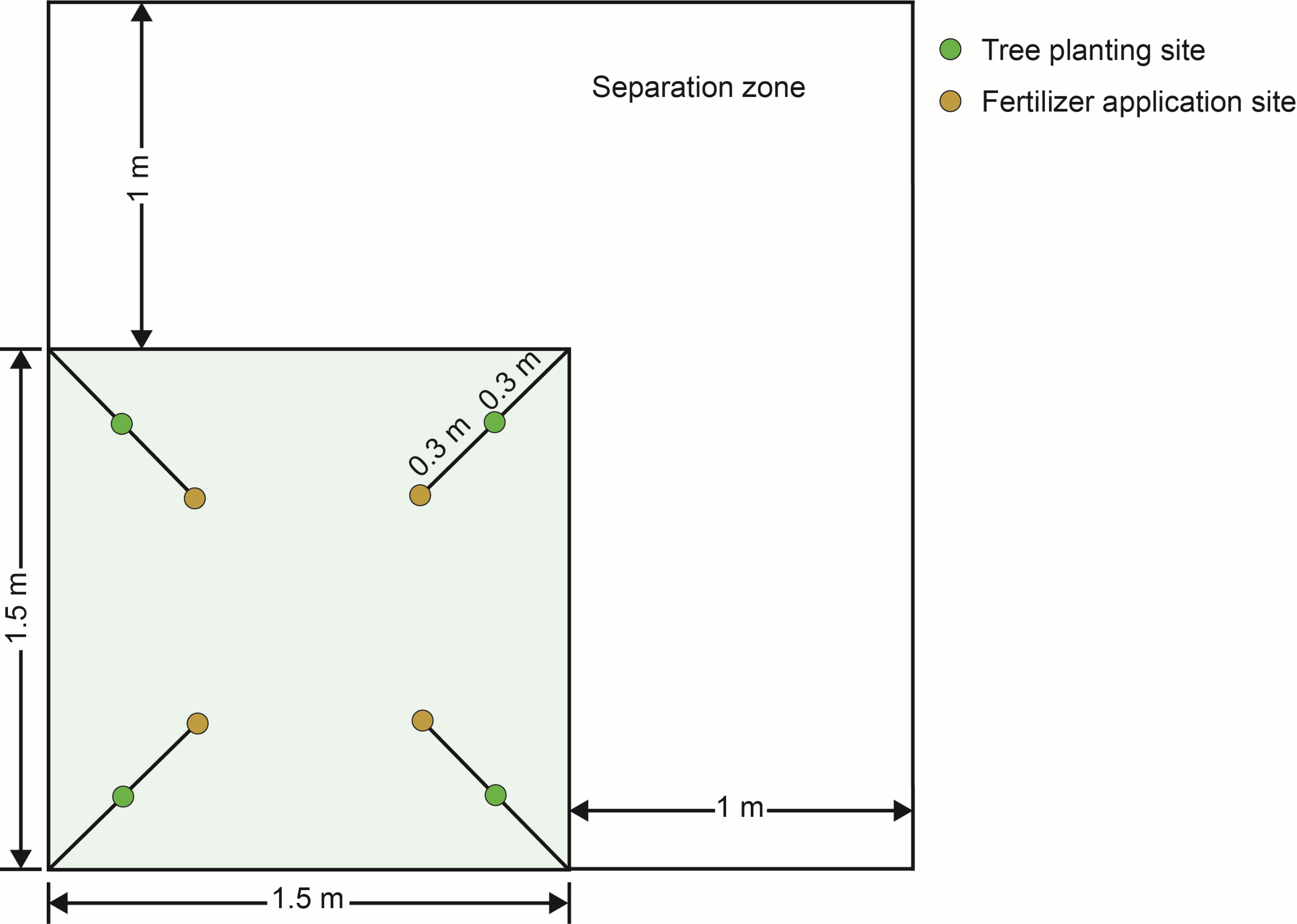


**Fig. S2** Plot size and interval distance (showing planting and fertilizing locations for saplings).

**Fig. S3** Differences in twig length (L), basal diameter (BD), and stem mass (SM) of *Malus sieversii* under N and P additions. Different lowercase letters indicated significant difference at *P* < 0.05 among each nutrient addition group. “*” indicated significant N-P interaction under N and P additions at *P* < 0.05, “ns” indicate non-significant N-P interaction. L: N10, P2, N20P2, and N10P4; M: N20, P4, and N20P4; H: N40, P8, N20P8, and N40P4.

**Fig. S4** Differences in leaf area (LA), total petiole mass (TPM), total leaf area (TLA), total leaf mass (TLM), single petiole mass (SPM), and specific leaf area (SLA) of *Malus sieversii* under N and P additions. Different lowercase letters indicated significant difference at *P* < 0.05 among each nutrient addition group. “*” indicated significant N-P interaction under N and P additions at *P* < 0.05, “ns” indicate non-significant N-P interaction. L: N10, P2, N20P2, and N10P4; M: N20, P4, and N20P4; H: N40, P8, N20P8, and N40P4.


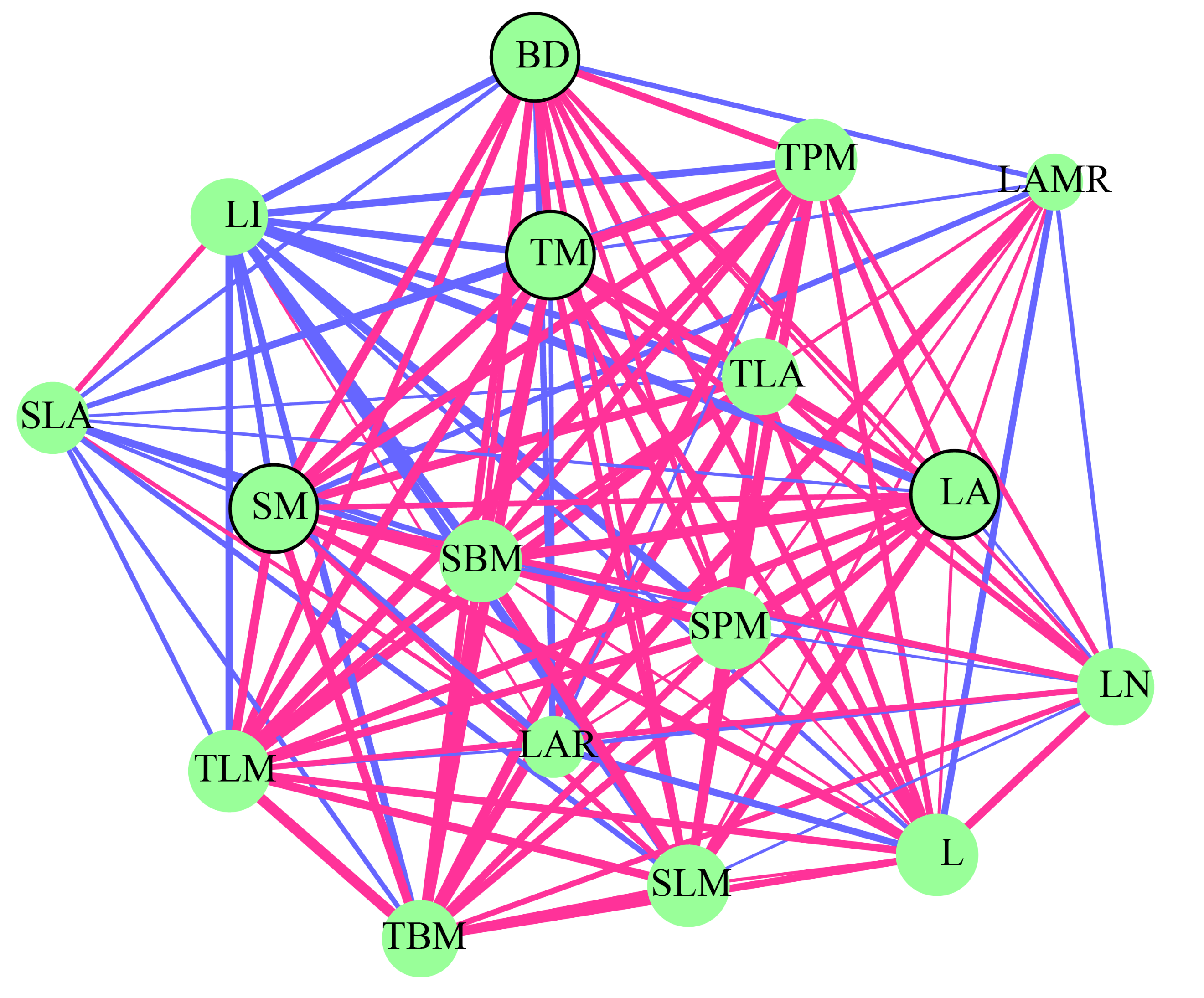


**Fig. S5** Plant Trait Network (PTN) of *Malus sieversii* for all data. Nodes represented different plant traits, and node size showed degree. Red and blue lines represented positive and negative correlations, respectively. Circles with black borders represent the key trait. The width of the line indicated the strength of the association. L: Length; BD: basal diameter; LA: leaf area; LN: leaf number; TM: twig mass; TBM: total blade mass; TPM: total petiole mass; SM: stem mass; TLA: total leaf area; TLM: total leaf mass; SPM: single petiole mass; SBM: single blade mass; SLM: single leaf mass; SLA: specific leaf area; LI: leaf intensity; LAR: leaf area ratio, and leaf area to stem mass ratio, LAMR.

**Supplementary Tables**

**Tables S1** Topological properties of plant trait networks of wild apple saplings under N and P additions.

| **Topological property** | **N** | **P** | **N20P*x*** | **N*x*P4** | **All data** |
| --- | --- | --- | --- | --- | --- |
| Edge number | 113 | 108 | 110 | 107 | 122 |
| Node number | 17 | 17 | 17 | 17 | 17 |
| Connectance | 0.831 | 0.794 | 0.809 | 0.787 | 0.897 |
| Average degree | 13.294 | 12.706 | 12.941 | 12.588 | 14.353 |
| Average path length | 1.169 | 1.206 | 1.191 | 1.213 | 1.103 |
| Edge connectivity | 6 | 6 | 8 | 6 | 10 |
| Clustering coefficient | 0.884 | 0.867 | 0.857 | 0.856 | 0.912 |
| Centralization betweenness | 0.019 | 0.028 | 0.019 | 0.030 | 0.005 |
| Centralization degree | 0.169 | 0.206 | 0.191 | 0.213 | 0.103 |
| Modularity | 0.057 | 0.068 | 0.059 | 0.078 | 0.022 |

**Tables S2** Node (plant trait) properties of plant trait networks of wild apple saplings under N and P additions.

| **Trait** | **N** | | **P** | | **N20P*x*** | | **N*x*P4** | | **All data** | |
| --- | --- | --- | --- | --- | --- | --- | --- | --- | --- | --- |
|  | **Degree** | **Betweenness** | **Degree** | **Betweenness** | **Degree** | **Betweenness** | **Degree** | **Betweenness** | **Degree** | **Betweenness** |
| L | 15 | 3.089 | 15 | 4.103 | 15 | 2.79 | 11 | 1.426 | 15 | 1.08 |
| BD | **16** | 3.541 | 15 | 3.836 | **16** | 3.63 | 15 | 4.292 | **16** | 1.37 |
| SM | **16** | 3.541 | **16** | 4.810 | 15 | 2.79 | **16** | 5.106 | **16** | 1.37 |
| TBM | 14 | 0.771 | 14 | 1.265 | 14 | 1.16 | 14 | 1.530 | 14 | 0.24 |
| TPM | 14 | 0.771 | 14 | 1.265 | 14 | 1.16 | 15 | 2.590 | 15 | 0.71 |
| TLM | 14 | 0.771 | 14 | 1.265 | 14 | 1.16 | 15 | 2.590 | 15 | 0.71 |
| TM | 15 | 2.871 | **16** | 4.810 | 15 | 2.37 | **16** | 5.106 | **16** | 1.37 |
| LN | 10 | 1.344 | 9 | 1.395 | 10 | 1.29 | 10 | 1.326 | 14 | 0.89 |
| SBM | 14 | 0.746 | 13 | 0.291 | 14 | 1.68 | 12 | 0.091 | 15 | 0.90 |
| SPM | 14 | 0.746 | 13 | 0.291 | 13 | 0.42 | 12 | 0.091 | 15 | 0.90 |
| SLM | 14 | 0.746 | 13 | 0.291 | 14 | 1.68 | 12 | 0.091 | 15 | 0.90 |
| TLA | 14 | 0.771 | 13 | 0.808 | 13 | 0.57 | 13 | 1.171 | 14 | 0.24 |
| LA | 14 | 0.746 | 12 | 0.000 | 13 | 1.01 | 12 | 0.091 | **16** | 1.37 |
| SLA | 13 | 0.669 | 11 | 0.929 | 10 | 0.88 | 12 | 0.917 | 13 | 0.47 |
| LI | 13 | 0.077 | 14 | 1.586 | 14 | 1.64 | 14 | 1.530 | 14 | 0.55 |
| LAR | 6 | 0.100 | 8 | 0.944 | 8 | 1.00 | 9 | 0.951 | 11 | 0.67 |
| LAMR | 10 | 1.696 | 6 | 0.111 | 8 | 0.79 | 6 | 0.100 | 10 | 0.27 |

L: Length; BD: basal diameter; LA: leaf area; LN: leaf number; TM: twig mass; TBM: total blade mass; TPM: total petiole mass; SM: stem mass; TLA: total leaf area; TLM: total leaf mass; SPM: single petiole mass; SBM: single blade mass; SLM: single leaf mass; SLA: specific leaf area; LI: leaf intensity; LAR: leaf area ratio; leaf area to stem mass ratio, LAMR. *n* = 109, 76, 76, and 65, respectively.
